# The E3 ubiquitin ligase TRIM56 promotes aggregation and activation of Src protein through Lys63-linked polyubiquitination in hepatocellular carcinoma

**DOI:** 10.1038/s41419-025-08074-1

**Published:** 2025-10-16

**Authors:** Lihui Zhu, Xiuling Cui, Hongwei Xu, Min Yang, Lihui Han

**Affiliations:** 1 Department of Immunology, Shandong Provincial Key Laboratory of Infection & Immunology, Shandong University School of Basic Medical Sciences, Jinan, Shandong China; 2 Department of Gastroenterology, Shandong Provincial Hospital Affiliated to Shandong First Medical University, Jinan, Shandong China

**Keywords:** Ubiquitylation, Liver cancer, Liver cancer

## Abstract

Elevated activity of proto-oncogene tyrosine kinase Src is associated with tumorigenesis and progression of hepatocellular carcinoma (HCC). It is well recognized that activation of Src is mainly driven by its intermolecular autophosphorylation. However, the precise mechanism involved in the activation of Src remains to be fully understood. Here we identified tripartite motif-containing protein (TRIM) 56, a member of E3 ubiquitin ligase family, as a novel regulator of Src activation. The data revealed that TRIM56 directly interacted with Src and catalyzed the polyubiquitination and subsequent aggregation of Src, resulting in Src activation and HCC progression. Mechanistically, TRIM56 interacted with the SH3 domain of Src protein via its B-box1 domain and catalyzed the Lys63-linked polyubiquitination of Src at the Lys184 residue, leading to the aggregation and activation of Src. Altogether, here we demonstrated that TRIM56 acted as a tumor promoter in HCC and it exerted a novel regulatory effect on Src activation. Thus, this study suggested a promising therapeutic strategy for HCC patients by targeting TRIM56.

## Introduction

The proto-oncogene tyrosine kinase Src is ubiquitously expressed and highly activated in a lot of human malignancies, including hepatocellular carcinoma (HCC) [1, 2]. It plays a crucial role in numerous signal transduction pathways, ranging from cell proliferation to metastasis [3, 4]. Src consists of a myristoylated N-terminal segment, a Src homology (SH)3 domain, an SH2 domain, and it is tightly regulated by two phosphorylation events. When the phosphorylation occurs at the Tyrosine(Tyr) 419 site, Src undergoes a conformational change, so that phosphorylation of one Src molecule by another at the Tyr419 residue could permit positive autoactivation. However, when Src is phosphorylated at tyrosine 530, its kinase activity is blocked [5, 6]. Thus, Src activation is mainly driven by the intermolecular autophosphorylation and a positive kinase activation cascade [7].

Previous studies have reported that the activation of Src is regulated by ubiquitination. It is reported that the carboxyl terminus of constitutive heat shock cognate 70-interacting protein (CHIP) catalyzes the K63-linked polyubiquitination of Src, leading to its recruitment and activation [8]. Moreover, the E3 ubiquitin ligase tripartite motif-containing (TRIM) 50 and TRIM7 induce the K48-linked polyubiquitination and proteasomal degradation of Src, thereby inhibiting the progression of ovarian cancer and HCC, respectively [9, 10]. Besides, Casitas B-lineage lymphoma (C-CBL) also acts as an E3 ligase and catalyzes the neddylation of Src, which further induces the polyubiquitination and proteasome-mediated degradation of Src [11].

Aberrant Src activity is often associated with the malignant progression of human cancers [12]. Several Src inhibitors, such as bosutinib, dasatinib, ponatinib, and vandetanib, have been developed and applied for the treatment of cancers with Src overexpression, demonstrating a great potential of regulating oncogenic Src in clinical treatment [13]. Despite the great progress in the development of Src inhibitors, the prognosis of Src over-expressing cancer patients still remains poor, indicating the urgent need of developing therapeutic strategies by targeting Src. Therefore, deciphering the regulatory mechanism of Src to facilitate the discovery of novel Src inhibitor might provide a great therapeutic potential for efficient manipulation of cancer.

TRIM protein family consists of a variety of ubiquitin E3 ligases, including 11 subfamilies and more than 100 known proteins up to now [14]. The TRIM proteins are involved in various cellular processes, including carcinogenesis, innate immunity, autophagy, and signal transduction [15]. TRIM56 is identified as a member of the fifth subfamily of TRIM proteins, most of which are newly identified E3 ubiquitin proteins playing extensive roles in physiological and pathological processes, such as carcinogenesis, neurological diseases, innate immune signaling, antiviral activity, and genetic disorders.

The role of TRIM56 in innate immunity has been well defined [16, 17]. It is reported that TRIM56 can be induced by viral infection, resulting in the production of type I interferon (IFN-I) [18]. TRIM56 exerts antiviral effect against distinct positive-sense single-stranded RNA viruses, and it is also effective against negative-sense single-stranded RNA viruses [19]. TRIM56 acts as an E3 ubiquitin ligase and promotes the Lys63-linked ubiquitination of stimulator of interferon genes (STING), leading to the dimerization of STING and the enhanced production of cytosolic DNA-induced type I IFN [20]. Besides, TRIM56 induces monoubiquitination of cyclic GMP-AMP Synthase (cGAS), which results in its stable dimerization, followed by the enhancement of its DNA-binding activity [21]. Meanwhile, TRIM56 plays a novel role in the antiviral innate immunity by positively regulating the Toll-like receptor (TLR)3-mediated antiviral signaling pathway [22].

Despite the recognized effect of TRIM56 in innate immunity, its role in human malignancies remains to be fully understood. In the development of breast cancer, TRIM56 stabilizes the estrogen receptor alpha protein through the K63-linked polyubiquitination, leading to the regulation of estrogen signaling and further cancer progression [23]. Another study reports that TRIM56 acts as an E3 ubiquitin ligase to induce the ubiquitination and degradation of vimentin protein, leading to the attenuation of ovarian cancer progression [24]. It is reported that TRIM56 suppresses multiple myeloma progression by activating TLR3 signaling [25]. Besides, TRIM56 could regulate the forkhead box M1-mediated DNA repair and reduce the radiosensitization of human glioblastoma [26]. Present studies suggest that TRIM56 is a vital regulator in human malignancies, thus we are interested in defining whether TRIM56 plays a role in HCC.

In this study, our data revealed that TRIM56 enhanced the protein stability of Src and promoted Src activation by interacting with the SH3 domain of Src via its B-box1 domain. Moreover, TRIM56 induced the Lys63-linked polyubiquitination of Src at the Lys184 residue via its RING domain, and further promoted the progression of HCC by regulating the aggregation and activation of Src. In summary, our study demonstrated a novel regulatory effect of TRIM56 on Src activation in HCC progression, which might pave a new avenue for the development of therapeutic strategies for HCC.

## Results

### TRIM56 interacted with the SH3 domain of Src protein via its B-box1 domain

**Fig. 1:**
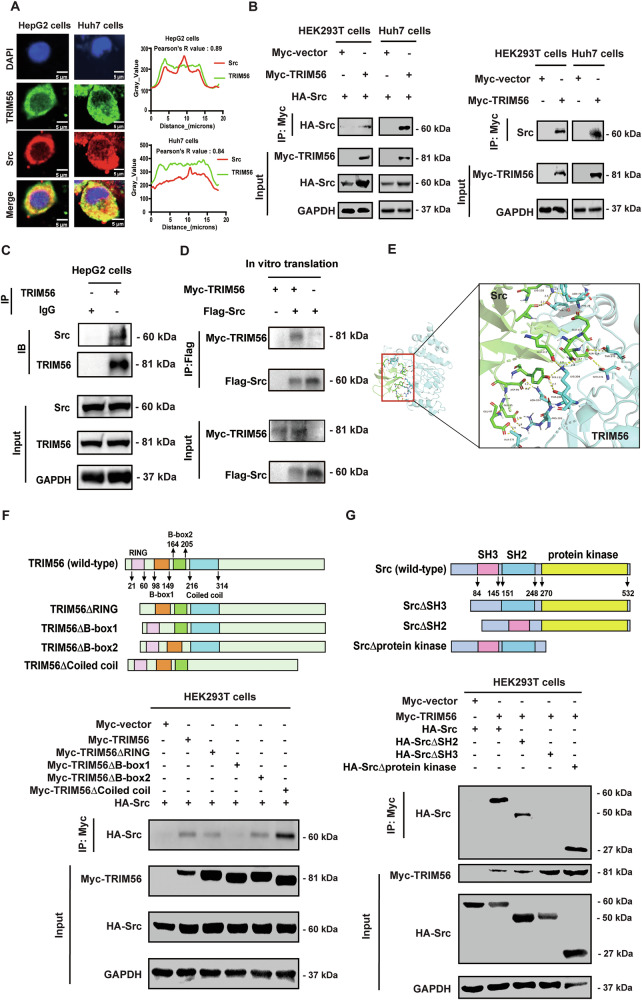
TRIM56 interacted with the SH3 domain of Src protein via its B-box1 domain. **A** HCC cells were fixed and stained with the primary antibodies against TRIM56 and Src, followed by the staining with the fluorescence-conjugated secondary antibodies. DAPI was used to stain the nuclei, and the co-localization of TRIM56 (green) and Src (red) was visualized as yellow fluorescence in the merged panel. The co-localization of TRIM56 and Src was calculated by the Pearson’s correlation coefficient, and represented as *R* Value in the Figure (0.5 < R value <1 is regarded as colocalization of the proteins). Scale bar, 5 μm. **B** HEK293T cells and Huh7 cells were co-transfected with Myc-TRIM56 and HA-Src plasmids (left panel) or only transfected with Myc-TRIM56 plasmid (right panel). The interaction between Myc-TRIM56 and the exogenous or endogenous Src proteins was detected by immunoprecipitation. **C** The endogenous interaction between TRIM56 and Src was detected by IP assay. **D** The TRIM56 and Src proteins were obtained from an in vitro transcription and translation system, and their direct interaction was detected by co-IP assay. **E** Molecular docking model illustrating the interaction interface between TRIM56 and Src. PDB: TRIM56: 5JW7(blue), Src: 8BQ3(green). **F** The TRIM56 plasmid or its domain deleted mutants together with a HA-Src plasmid were co-transfected into HEK293T cells. The binding between Myc-TRIM56 mutants and HA-Src was detected by co-IP. **G** HEK293T cells were co-transfected with a Myc-TRIM56 plasmid together a HA-Src plasmid or its structural domain-deleted mutants. The interaction between Myc-TRIM56 and HA-Src mutants was detected by co-IP. The presented figures are representative data from at least three independent experiments.

Accumulating evidence has demonstrated that TRIM proteins typically exert their biological effect by regulating tumor promoters or tumor suppressors [27]. To explore whether TRIM56 could interact with Src, we performed the immunofluorescence microscopy assay in Huh7 cells and HepG2 cells to examine the expression and subcellular localization of TRIM56 and Src. Co-localization of TRIM56 and Src was confirmed in both the Huh7 and HepG2 cells by immunofluorescence microscopy assay (Fig. 1A). Subsequently, we conducted the co-immunoprecipitation (co-IP) assay, and the data suggested that TRIM56 could interact with both the exogenous and endogenous Src (Fig. 1B, C). Furthermore, we obtained TRIM56 and Src proteins by an in vitro transcription and translation system, and the IP assay further demonstrated that TRIM56 could directly interact with Src in vitro (Fig. 1D). Additionally, we simulated the spatial binding between TRIM56 and Src by analyzing their interaction with the ZDOCK SERVER platform (https://zdock.umassmed.edu/), which verified their spatial physicochemical interaction (Fig. 1E). Altogether, these data indicated that TRIM56 directly interacted with Src protein.

To further elucidate the interacting domains of TRIM56 and Src, we generated a series of domain-deleted mutants of both TRIM56 and Src. TRIM56 consists of a RING domain, two B-box domains and a coiled-coil domain (Fig. 1F), while Src consists of an SH3 domain, an SH2 domain and a protein kinase domain (Fig. 1G). We co-transfected HEK293T cells with the HA-Src plasmid and the TRIM56 mutant constructs, followed with the co-IP assay to define their interaction. The data revealed that the B-box1 domain-deleted mutant of TRIM56 could not interact with Src protein, indicating that the B-box1 domain of TRIM56 was responsible for the interaction of TRIM56 and Src (Fig. 1F). Further investigation revealed that when the SH3 domain of Src was deleted, the interaction between Src and TRIM56 was abolished (Fig. 1G), indicating the SH3 domain of Src was the interacting domain with TRIM56. All these data suggested that the B-box1 domain of TRIM56 and the SH3 domain of Src were the molecular basis of the interaction between TRIM56 and Src.

### TRIM56 promoted the abundance and activation of Src

**Fig. 2:**
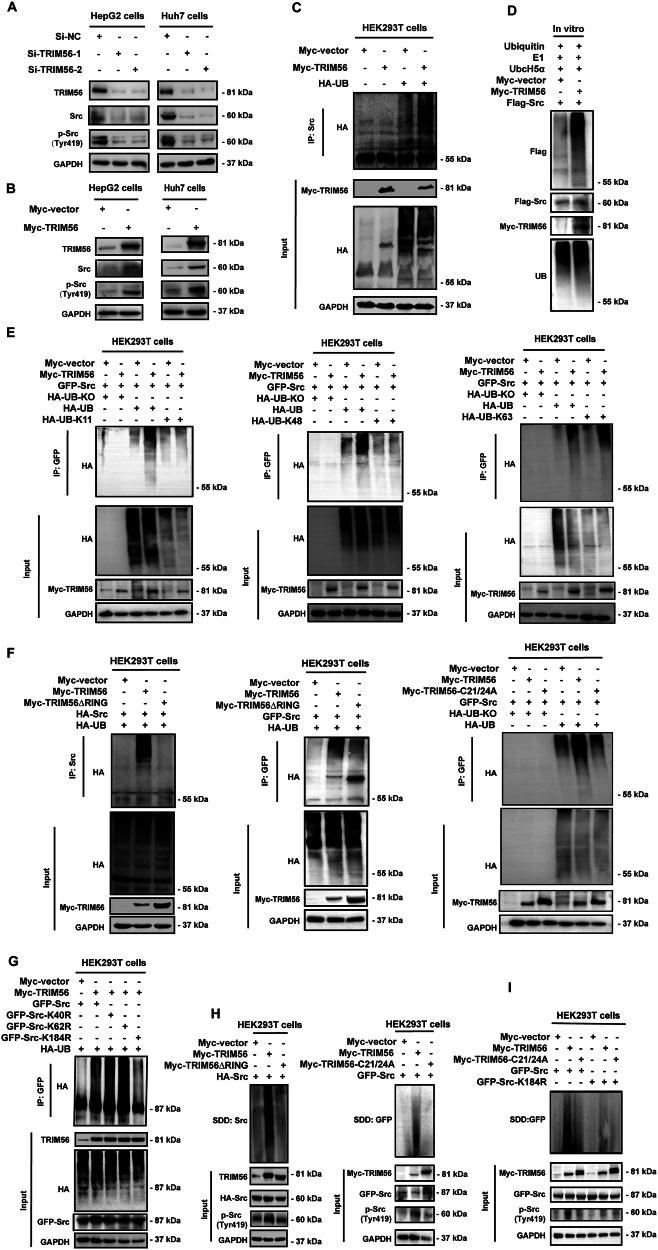
TRIM56 promoted the aggregation and activation of Src via inducing Lys63-linked polyubiquitination of Src at the Lys184 residue. **A** HepG2 and Huh7 cells were transfected with Si-RNA specifically targeting TRIM56 (Si-TRIM56-1 or Si-TRIM56-2) or its nonsense control (Si-NC), and the protein levels of Src and p-Src (Tyr419) were detected by western blot. **B** HepG2 and Huh7 cells were transfected with Myc-TRIM56 plasmid, and the protein levels of Src and p-Src (Tyr419) were detected by western blot. **C** HEK293T cells were co-transfected with Myc-TRIM56 plasmid and HA-UB plasmid, and the ubiquitination status of Src was analyzed by co-IP. **D** In vitro ubiquitination analysis of the ubiquitination of Src catalyzed by TRIM56 at the presence of in vitro translated Myc-TRIM56 and Flag-Src, E1, UbcH5a, and ubiquitin. **E** HEK293T cells were co-transfected with Myc-TRIM56 plasmid, GFP-Src plasmid, together with the indicated HA-UB mutants. The ubiquitination status of Src was analyzed by co-IP. **F** HEK293T cells were co-transfected with HA-Src or GFP-Src plasmid, together with HA-UB plasmid, Myc-TRIM56 plasmid or Myc-TRIM56 mutant (Myc-TRIM56ΔRING, Myc-TRIM56-C21/24A) plasmid, and the ubiquitination status of Src was analyzed by co-IP. **G** HEK293T cells were co-transfected with the Myc-TRIM56 plasmid, and the HA-UB plasmid, GFP-Src plasmid or the GFP-Src site mutant (GFP-Src-K40, GFP-Src-K62, or GFP-Src-K184), and the ubiquitination status of GFP-Src was analyzed by co-IP. **H** HEK293T cells were co-transfected with HA-Src or GFP-Src plasmid, together with the Myc-TRIM56 plasmid or Myc-TRIM56 mutant (Myc-TRIM56ΔRING or C21/24A); and the aggregation of Src was measured by SDD-AGE. **I** HEK293T cells were co-transfected with GFP-Src or GFP-Src-K184R plasmid, together with the Myc-TRIM56 plasmid or the Myc-TRIM56 mutant (Myc-TRIM56-C21/24A), and the aggregation of Src was measured by SDD-AGE. The presented figures are representative data from at least three independent experiments.

We have defined that TRIM56 directly interacts with Src protein, and we are further interested in defining whether TRIM56 has any regulatory effect on Src. Thus, we constructed the loss-of-function and gain-of-function of HCC cellular models by transfecting the small interference RNAs against TRIM56 (Si-TRIM56-1 and Si-TRIM56-2) or transfecting the TRIM56 expression plasmid into HCC cells, respectively. Then the successful knockdown and overexpression of TRIM56 were validated by western blot. Our data revealed that after the successful knockdown of TRIM56 by Si-TRIM56, the protein levels of both Src and p-Src were significantly reduced (Fig. 2A). Moreover, in the TRIM56 over-expression cellular models, the protein levels of both Src and p-Src were significantly increased (Fig. 2B). The above data implied that TRIM56 significantly increased the protein level of Src and further enhanced its phosphorylation and activation.

We further investigated whether TRIM56 could regulate the mRNA level of Src by real-time polymerase chain reaction (PCR) in the loss-of-function and gain-of-function HCC cellular models. Our data revealed that the overexpression of TRIM56 had no effect on the mRNA level of Src at all the detected dosages or time points (Supplementary Fig. S1A, B). Similarly, knockdown of TRIM56 had no effect on the mRNA level of Src (Supplementary Fig. S1C). Next, we constructed the enzymatic activity-dead TRIM56 mutant(C21/24A) by replacing the Cysteine 21 and Cysteine 24 residues with arginine. We further performed the protein stability assay after the de novo protein synthesis in the TRIM56 or its mutant (C21/24A) overexpressing HCC cells was blocked by cycloheximide (CHX). The data suggested that TRIM56 upregulated the protein level of Src, while the increased Src protein abundance was rescued in the TRIM56 mutant (C21/24A) overexpressing HCC cells (Supplementary Fig. S1D). Altogether, these data suggested that TRIM56 could promote the protein stability and activation of Src.

### TRIM56 induced the Lys63-linked polyubiquitination of Src at the Lys184 residue

Considering that TRIM56 is one of the RING-type E3 ubiquitin ligase, we then tried to determine if TRIM56 could induce the ubiquitination of Src. The ubiquitination assay revealed that the polyubiquitin chain could be conjugated to Src protein by TRIM56 (Fig. 2C). We also performed an in vitro ubiquitination assay, and the data revealed that Src was markedly polyubiquitinated in the presence of TRIM56 in a cell-free ubiquitination reaction (Fig. 2D). To further define the ubiquitination type of Src mediated by TRIM56, we constructed the HA-tagged dominant-negative ubiquitin mutant (UB-KO) by the substitution of all lysine residues with arginine. We further construct the ubiquitin Lys11 (UB-K11), ubiquitin Lys48 (UB-K48) and ubiquitin Lys63 (UB-K63) expression plasmids by the substitution of lysine with arginine except the indicated sites. Further ubiquitination assay data revealed that TRIM56 could induce the K63-linked polyubiquitination of Src protein (Fig. 2E). To further validate the ubiquitination of Src catalyzed by TRIM56, we then performed the ubiquitination assay of the TRIM56 RING domain truncated construct or the TRIM56 mutant(C21/24A) transfected cells. Ubiquitination assay data revealed that the polyubiquitination of Src was significantly reduced when the RING domain deleted mutant or the enzymatic activity dead mutant of TRIM56 was transfected (Fig. 2F). Thus, these data indicated that both the RING domain and the E3 ligase enzymatic activity site of TRIM56 were required for it to catalyze the polyubiquitination of Src.

Next, we are interested in defining the lysine residues of Src responsible for the TRIM56-mediated polyubiquitination. The potential ubiquitin-conjugating lysine sites in Src protein were predicted using the ubiquitin site prediction website (http://ubpred.org). The screening data revealed that the Lys40, Lys62, and Lys184 were the lysine sites with high probability of ubiquitination. Thus, we generated the site mutants of Src including the Src-K40R, Src-K62R, and Src-K184R mutants, in which the lysine residue at the position 40, 62, or 184 in Src was replaced with arginine. The following ubiquitination assay revealed that when the Src Lys184 residue was replaced, TRIM56 failed to conjugate the polyubiquitin chain to Src, which indicated that the Src Lys184 residue was responsible for the ubiquitination catalyzed by TRIM56 (Fig. 2G). Further immunofluorescence microscopy assay demonstrated that the co-localization of TRIM56 and Src were significantly diminished in the Src K184R mutant or the SH3 domain-deleted mutant transfected cells (Supplementary Fig. S2A). Taken together, these data demonstrated that TRIM56 induced the Lys63-linked polyubiquitination of Src at the Src Lys184 residue via its RING domain.

### TRIM56 promoted aggregation of the Src protein

Since TRIM56 could promote the activation of Src, we are further interested in investigating the mechanism involved in this process. Src undergoes an intermolecular autophosphorylation at the Tyr419 site catalyzed by another Src molecule in the activation loop, which further increases the kinase activity. Hence, we speculated that the promoted Src activity might be the consequence of Src aggregation. Subsequently, the aggregation of Src induced by TRIM56 was measured by the semi-denaturing detergent agarose-gel electrophoresis (SDD-AGE). The data revealed that TRIM56 could significantly induce the aggregation of Src. Moreover, both the RING domain-deleted mutant and the enzymatic activity-dead TRIM56 mutant failed to mediate the aggregation of Src (Fig. 2H). Further investigation revealed that the K184R mutant of Src significantly abolished TRIM56-induced Src aggregation (Fig. 2I), indicating that the Lys184 residue was a functional ubiquitination site, and the aggregation of Src was induced by the TRIM56-mediated Lys63-linked polyubiquitination.

### TRIM56 acted as a tumor promoter in HCC cells

**Fig. 3:**
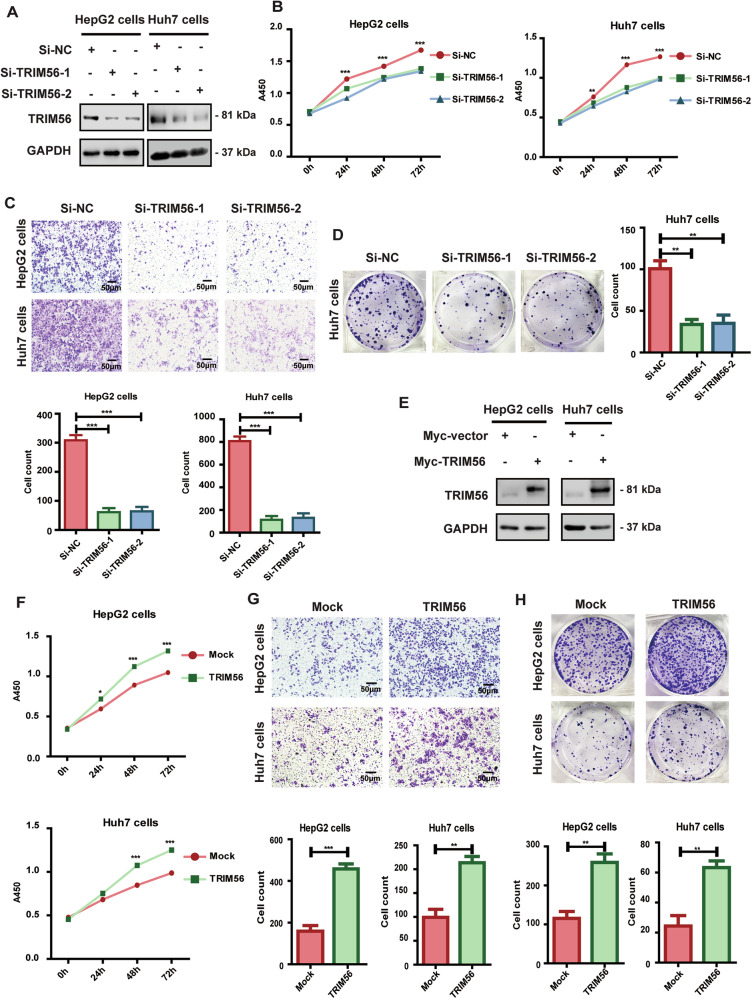
TRIM56 acted as a tumor promoter in HCC cells. **A**–**D** HepG2 and Huh7 cells were transfected with Si-NC, or Si-TRIM56; and the successful knockdown of TRIM56 was confirmed by western blot (**A**). The proliferation (**B**), invasion (**C**), and colony formation (**D**) of the transfected HCC cells were further detected and analyzed. **E**–**H** HepG2 and Huh7 cells were transfected with Myc-TRIM56 plasmid, and the successful overexpression of TRIM56 was confirmed by western blot assay (**E**). The proliferation (**F**), invasion (**G**), and colony formation (**H**) of the transfected HCC cells were further detected and analyzed. Scale bar, 50 μm. *P < 0.05, **P < 0.01, and ***P < 0.001 (Two-way ANOVA or Student’s *t* test) for statistical analysis of the indicated groups, error bars represent mean ± SD. The presented figures are representative data from at least three independent experiments.

We have demonstrated that TRIM56 promoted the aggregation and activation of Src, thus we wondered whether TRIM56 had any regulatory effect on HCC cells. We investigated the effect of TRIM56 on HCC by using the loss-of-function and the gain-of-function HCC cellular models. Our data revealed that the proliferation, invasion, and colony formation capabilities of the TRIM56-knockdown HCC cells were significantly inhibited (Fig. 3A–D), while the proliferation, invasion, and colony formation capabilities of the TRIM56-overexpressing HCC cells were significantly promoted (Fig. 3E–H). Altogether, these data indicated that TRIM56 acted as a tumor promoter and promoted the malignant behaviors of HCC cells.

### TRIM56 promoted HCC via its upregulation of Src

**Fig. 4:**
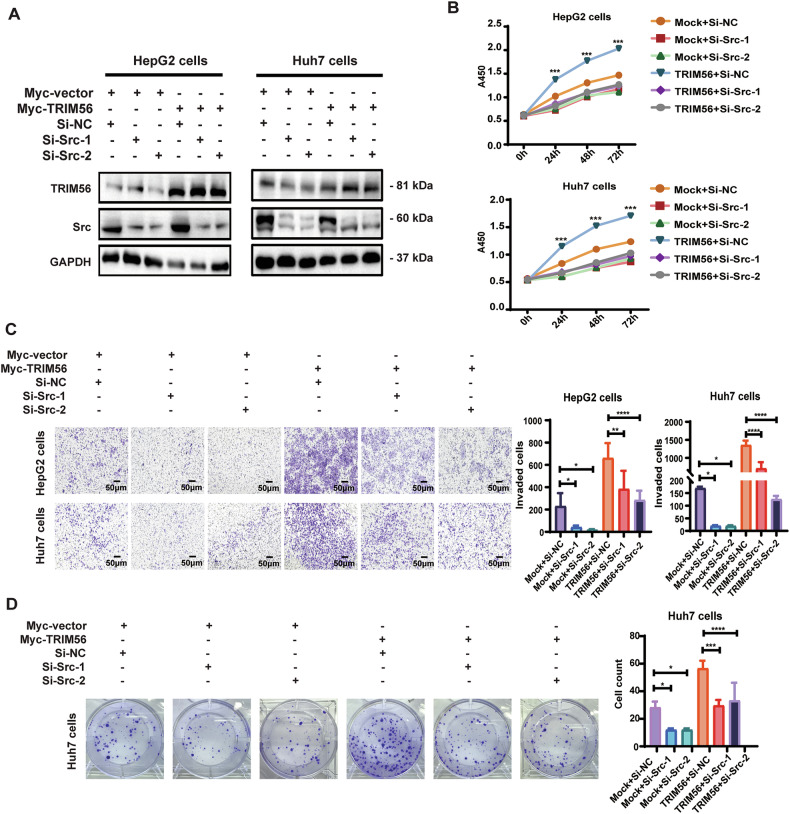
TRIM56 promoted HCC through the upregulation of Src. **A**–**D** HepG2 and Huh7 cells were transfected with Myc-TRIM56 plasmid together with Si-RNA specifically targeting Src (Si-Src-1 or Si-Src-2) or its nonsense control (Si-NC); and the protein levels of TRM56 and Src were detected by western blot (**A**). The proliferation (**B**), invasion (**C**), and colony formation (**D**) of the transfected HCC cells were further detected and analyzed. Scale bar, 50 μm.*P < 0.05, **P < 0.01, ***P < 0.001 and ****P < 0.0001 (Two-way ANOVA), for statistical analysis of the indicated groups, error bars represent mean ± SD. The presented figures are representative data from at least three independent experiments.

**Fig. 5:**
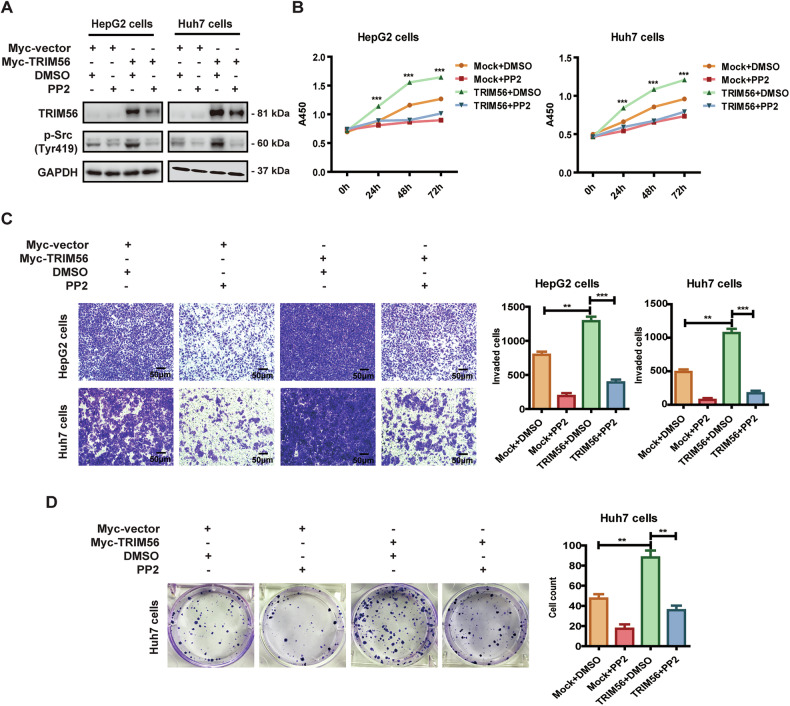
TRIM56 promoted the malignant behaviors of HCC cells through its activation of Src. **A**–**D** HepG2 and Huh7 cells were transfected with Myc-TRIM56 plasmid before the treatment with PP2 (10 μM). The cells were further cultured for 24 h, and the protein levels of TRIM56 and p-Src (Tyr419) were detected by western blot (**A**). The proliferation (**B**), invasion (**C**), and colony formation (**D**) of the transfected HCC cells were further detected and analyzed. Scale bar, 50 μm. **P < 0.01 and ***P < 0.001 (Two-way ANOVA or Student’s *t* test) for statistical analysis of the indicated groups, error bars represent mean ± SD. The presented figures are representative data from at least three independent experiments.

In order to define whether TRIM56 exerted its oncogenic role in HCC cells through its upregulation of Src, we transfected the TRIM56-overexpressing HCC cells with small interference RNAs against Src (Si-Src-1 and Si-Src-2) for further analysis of the malignant behaviors of HCC cells. The data revealed that knockdown of Src significantly rescued the TRIM56-promoted proliferation, invasion, and colony formation of HCC cells (Fig. 4A–D). Similarly, when the activation of Src in these TRIM56-overexpressing HCC cells was inhibited by its specific inhibitor PP2, the proliferation, invasion, and colony formation of these cells were all dramatically inhibited (Fig. 5A–D). These data suggested that TRIM56 acted as a tumor promoter in HCC through the upregulation and activation of Src.

### TRIM56 exerted its oncogenic effect on HCC cells through its functional domains

**Fig. 6:**
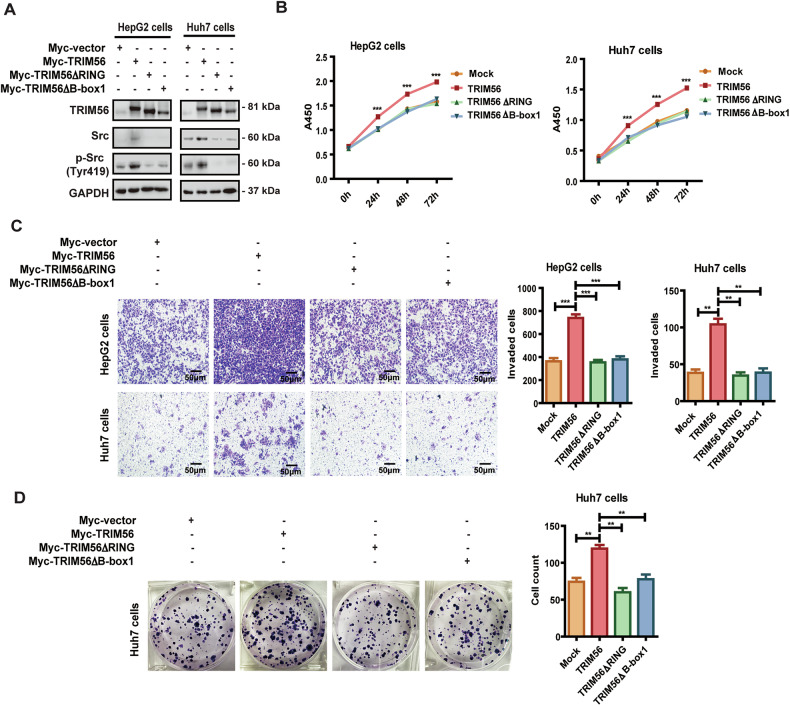
TRIM56 exerted its oncogenic effect on HCC cells through its RING domain and B-box1 domain. **A**–**D** HepG2 and Huh7 cells were transfected with Myc-TRIM56 plasmid or TRIM56 truncation mutant (Myc-TRIM56ΔRING or Myc-TRIM56ΔB-box1), and the successful overexpression of TRIM56 or its truncation mutants in the transfected cells was detected by western blot. The protein levels of Src and p-Src in the transfected cells were detected by western blot (**A**). The proliferation (**B**), invasion (**C**), and colony formation (**D**) of these transfected HCC cells were further detected and analyzed. Scale bar, 50 μm. **P < 0.01 and ***P < 0.001 (Two-way ANOVA or Student’s *t* test) for statistical analysis of the indicated groups, error bars represent mean ± SD. The presented figures are the representative data from at least three independent experiments.

We have defined that the RING domain was responsible for the ubiquitination and aggregation of Src induced by TRIM56, and the B-box1 domain was indispensable for the interaction between TRIM56 and Src. Then we further investigated whether TRIM56 exerted its oncogenic effect on HCC cells through these two functional domains. The data revealed that deletion of either the RING domain or the B-box1 domain in TRIM56 significantly abolished the upregulation and activation of Src mediated by TRIM56, which indicated that TRIM56 upregulated and activated Src through these two functional domains (Fig. 6A). Furthermore, both the RING domain-deleted mutant and the B-box1 domain-deleted mutant could significantly abolish the tumor promoter effect of TRIM56 on HCC cells (Fig. 6B–D). Taken together, these data indicated that both the RING domain and the B-box1 domain were necessary for TRIM56 to exert its oncogenic effect through regulating Src in HCC cells.

### TRIM56 was overexpressed in clinical HCC tissues

**Fig. 7:**
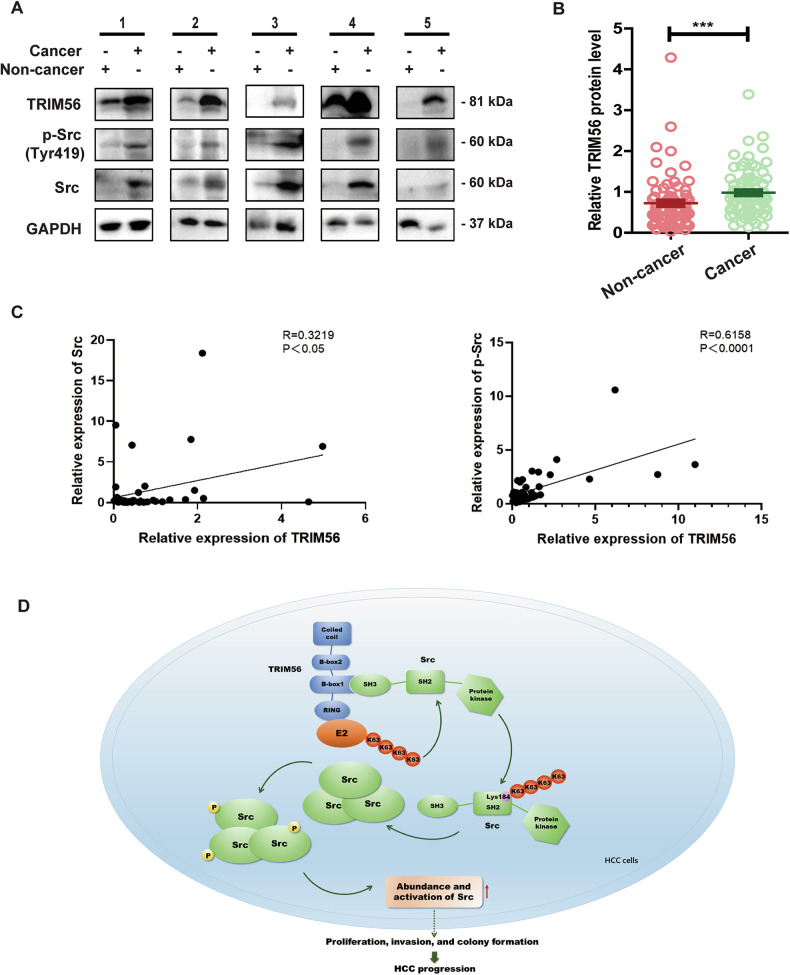
TRIM56 was overexpressed in the clinical HCC tissues. **A** Protein level of TRIM56, p-Src and Src in the HCC tissues and corresponding noncancerous liver tissues from clinical HCC patients were detected by western blot. Presented images are the representative blots from the investigated HCC patients. **B** Statistical analysis of TRIM56 expression by western blot assay in the HCC tissues and corresponding noncancerous liver tissues from the investigated HCC patients, *n* = 75. **C** The correlation of TRIM56 and Src or p-Src expression in the HCC tissues from the investigated HCC patients, *n* = 27. **D** Schematic illustration of the interaction between TRIM56 and Src protein, as well as the potential mechanism. ***P < 0.001 (Student’s *t* test) for statistical analysis of the indicated groups, error bars are mean ± SD.

Our data have identified TRIM56 as a tumor promoter in HCC cells, then we further try to explore the expression level of TRIM56 in clinical HCC tissues and corresponding distal noncancerous liver tissues. The data revealed that the protein level of TRIM56 was increased in HCC tissues in comparison with the corresponding distal noncancerous liver tissues (Fig. 7A, B). To validate the positive regulation of Src activation by TRIM56, we analyzed the correlation between the level of TRIM56 and Src. The statistically analyzed data revealed that the level of TRIM56 was significantly positively correlated with the levels of Src and p-Src (Fig. 7C). Thus, these data in the clinical investigation of HCC tissues further suggested that TRIM56 was upregulated in HCC tissues and it might promote HCC progression via its positive regulation of Src activation.

Altogether, our data demonstrated that TRIM56 directly interacted with Src via its B-box1 domain, and further induced K63-linked polyubiquitination of Src at the Lys184 residue via its RING domain. Thus, TRIM56 promoted the aggregation and activation of Src, and further promoted HCC progression (Fig. 7D).

## Discussion

As a non-receptor tyrosine kinase, the protein expression and kinase activity of Src are frequently upregulated in many human cancers. It has been reported that Src can regulate cell adhesion, invasion, proliferation, and apoptosis to promote the malignant phenotype of HCC, which further restricts the survival of HCC patients [28]. Many HCC patients are diagnosed at advanced stages without any opportunity of feasible therapies including hepatectomy, radiofrequency ablation, and liver transplantation [29]. Thus, deciphering the precise regulatory mechanism of Src to facilitate the development of efficient therapeutic strategy for HCC is in urgent need. In this study we identified TRIM56 as a novel Src regulator and demonstrated that it directly interacted with Src and promoted Src activation in HCC, which indicated a potential therapeutic strategy for HCC by targeting TRIM56.

It is reported that the polyubiquitination of Src can modulate its recruitment and activation. Certain E3 ligases have been identified as regulators of Src polyubiquitination, such as CHIP, TRIM50, TRIM7, and C-CBL [9–11, 30]. In this study, we demonstrated that TRIM56 acted as an E3 ubiquitin ligase via its RING domain, and it catalyzed the K63-linked polyubiquitination of Src at the Lys184 residue, resulting in Src aggregation and further activation. Our data revealed that after the polyubiquitination of Src was catalyzed by TRIM56, the aggregation of Src was initiated. As a consequence, Src molecules became more affiliated in spatial position, and the intermolecular autophosphorylation among Src molecules was more efficient [6], leading to the significantly enhanced protein stability and activation of Src. Previous study identified ubiquitin as a new ligand to bind with a subset of SH3 domain [31], and defining the effect of the interaction between ubiquitin and SH3 domain of Src in the aggregation and activation process in HCC deserves further intensive investigation.

E3 ubiquitin ligases are usually involved in malignancies through modifying tumor promoters or suppressors. As an E3 ubiquitin ligase, TRIM56 functions as a pivotal regulator in the progression of certain human malignancies, such as breast cancer, ovarian cancer, and multiple myeloma [23–25]. However, the detailed precise regulatory mechanism of TRIM56 in cancer progression remains to be fully elucidated. Here we demonstrated that TRIM56 promoted the progression of HCC in cellular models and further validated it in clinical HCC tissues, which indicated that TRIM56 might be further defined as a potential therapeutic target for HCC. Our data revealed that TRIM56 directly interacted with Src via its B-box1 domain and further catalyzed the Lys63-linked polyubiquitination of Src at the Lys184 residue, resulting in Src aggregation and activation. Both the RING domain deleted mutant and the enzymatic dead mutant of TRIM56 abolished the ubiquitination and subsequent activation of Src, indicating that the effect of TRIM56 on Src activation was dependent on it enzymatic activity. When Src activation was inhibited by its specific Si-RNAs or inhibitor, the enhanced proliferation, invasion, and colony formation induced by TRIM56 was significantly rescued. Altogether, here we provided several evidences to demonstrate a novel regulation mode of TRIM56 on Src in the progression of HCC, which might provide promising therapeutic strategies for HCC patients.

In summary, here we identified the E3 ubiquitin ligase TRIM56 as a key regulator of Src activation as well as a pivotal promotor of HCC. Mechanically, TRIM56 directly interacted with Src and catalyzed the K63-linked polyubiquitination of Src at the Lys184 residue, resulting in the subsequent aggregation and activation of Src. Through this process, TRIM56 facilitated the progression of HCC. In all, targeting TRIM56 to inhibit Src activity might be a promising therapeutic strategy for HCC.

## Materials and methods

### Cell culture, SiRNAs and plasmids construction, and transfection

Human HCC cells including HepG2 and Huh7 cell lines, and human embryonic kidney (HEK)293T cells were obtained, and cultured as previously described [10]. All cell lines were cytogenetically tested and authenticated by STR profiling within 2 years. The small interfering RNAs targeting TRIM56 and Src were synthesized by RIBOBIO (RIBOBIO, Guangzhou, China). Gene-specific siRNA were listed as follows: Si-TRIM56-1 sense: 5′-GCAGACCCAACAAGAAGAATT-3′, and antisense : 5′-UUCUUCUUGUUGGGUCUGCTT-3′; Si-TRIM56-2 sense: 5′-CACGGCUCUAUCUCAUCAATT-3′, and antisense : 5′-UUGAUGAGAUAGAGCCGUGTT-3′; Si-Src-1 sense: 5′-GUUGUAUGCUGUGGUUUCATT-3’, and antisense : 5′-UGAAACCACAGCAUACAACTT-3′; Si-Src-2 sense: 5′-CAGUGUCUGACUUCGACAATT-3′, and antisense : 5′-UUGUCGAAGUCAGACACUGTT-3′. The TRIM56 plasmid was synthesized by OriGene (OriGene Technologies, Maryland, USA). All of the TRIM56 truncated plasmids, including the RING domain, B-box 1 domain, B-box 2 domain, or coiled-coil domain-deleted mutants and the enzymatic activity-dead (C21/24A) mutant, were generated by using the KOD-Plus-Mutagenesis kit (Toyobo, Osaka, Japan) according to the manufacture’s protocol. Src plasmid was synthesized by Vigene (Vigene Biosciences, Rockville, USA). The different domain-deleted mutants of Src were also constructed by the KOD-Plus-Mutagenesis kit. The different site mutants of Src including Src-K40R, Src-K62R, Src-K184R mutants were generated by the KOD-Plus-Mutagenesis kit and validated by gene sequencing. The HA-UB-KO plasmid was synthesized by MIAOLING (MIAOLING BIOLOGY, Hubei, China), and the source of HA-UB, HA-UB-K11, HA-UB-K48, and HA-UB-K63 was described before [32]. The transfections were performed as described previously[32].

### Cell proliferation, invasion, and colony formation assay

Proliferation, invasion, and colony formation capabilities of HepG2 and Huh7 cells were analyzed as previously described [33, 34].

### Western blot, co-IP, immunofluorescence, ubiquitination analysis, and SDD–AGE assay

Western blot, co-IP, immunofluorescence, and ubiquitination analysis were performed as described previously [32, 35, 36]. The SDD-AGE assay was carried out according to the published protocol [37]. The specific primary antibodies used in these assays included antibodies against TRIM56 (#ab154862, Abcam, Cambridge, MA, USA), Src (#11097-1-AP, 60315-1-Ig, Proteintech, Chicago, USA; #2109, Cell Signaling Technology, Beverly, USA), p-Src (#6943, Cell Signaling Technology, Beverly, USA), Myc (TA150121, OriGene Technologies, Maryland, USA), HA (#51064-2-AP, Proteintech, Chicago, USA), GFP (#66002-1-Ig, Proteintech, Chicago, USA), GAPDH (#6004-1-Ig, Proteintech, Chicago, USA), and β-actin (#TA-09, ZSGBBIO, Beijing, China).

### Real-time PCR assay

Total RNA was isolated from HCC cells and real-time PCR was performed as described before [32]. Primers for the human TRIM56 gene were forward: 5′- GAGCTCGAGCTGTTTCCCACGGGTCCTCGCCCTCC -3′, reverse: 5′- GATTCTAGAACTGTCCGGAGAACGGACCCGAAA -3′. Primers for the human Src gene were forward: 5′- GAGCGGCTCCAGATTGTCAA -3′ reverse: 5′- CTGGGGATGTAGCCTGTCTGT -3′. Relative mRNA levels of the genes were normalized to β-actin. Primers for the β-actin gene were forward: 5′- GGCACCACACCTTCTACAATG -3′, reverse: 5′- TAGCACAGCCTGGATAGCAAC -3′. The relative mRNA levels of target genes were obtained by using the 2^-ΔΔCt^ method, and all assays were performed in triplicate.

### In vitro binding and ubiquitination assay

The in vitro transcription and translation of Flag-Src and Myc-TRIM56 was performed by a TNT Quick Coupled Transcription/Translation System (Promega, Madison, WI, USA) according to the manufacturer’s protocol. The Flag-Src and Myc-TRIM56 proteins were expressed in vitro, mixed together, and analyzed by co-IP assay to determine the direct binding between TRIM56 and Src proteins.

For the in vitro ubiquitination assay, the mixture containing 2 µL of UBE1(5μm), 2 µL of UBcH5α(50μm), 10 µL of Myc-TRIM56, 10 µL of Flag-Src, 5 µL of Ubiquitin (1 mM), 5 µL of Mg-ATP, 5 µL of E3 Ligase Reaction Buffer(50 mM HEPES, pH 8.0, 50 mM NaCl, 1 mM TCEP), and nuclease-free water were mixed together to a final volume of 50 µL. The mixture was incubated in a 37 °C-water bath for 60 min, followed with the ubiquitination assay of Src.

### Clinical HCC specimens

75 pairs of matched specimens including the clinical HCC tissues and their corresponding noncancerous liver tissues (collected from the Department of Hepatobiliary Surgery, Shandong Provincial Hospital Affiliated to Shandong First Medical University) were used for the western blot assay to detect the protein level of TRIM56. The clinical and pathological characteristics of the collected HCC patients were shown in Table 1. Before the study, all the recruited patients signed the informed consents. All of the protocols met the ethical guidelines of the Helsinki Declaration and approved by the Medical Ethics Committee of Shandong Provincial Hospital.

**Table 1:** Clinicopathologic characteristics of HCC patients for western blot.

	HCC patients (*n* = 75, for western blot)
Characteristics	No. of patients (%)
Gender	
Male	50 (66.7%)
Female	25 (33.3%)
Age	
<54	28 (37.3%)
≥54	47 (62.7%)
AFP level	
<20 ng/ml	36 (48.0%)
≥20 ng/ml	39 (52.0%)
Liver cirrhosis history	
Yes	26 (34.7%)
No	49 (65.3%)
Tumor size	
<5 cm	42 (56.0%)
≥5 cm	33 (44.0%)
TNM stage	
I	36 (48.0%)
II	7 (9.3%)
III	18 (24.0%)
IV	14 (18.7%)
BCLC stage	
0	5 (6.7%)
A	32 (42.7%)
B	15 (20.0%)
C	19 (25.3%)
D	4 (5.3%)

### Statistical analysis

Statistical analysis was performed using GraphPad Prism 5 software (GraphPad, CA, USA). Quantitative variables were evaluated with two-tailed Student’s *t* test or one-way analysis of variance. *P* value < 0.05 was considered statistically significant, and the *P* value used in all analysis was two-tailed test.

## Supplementary information

Supplementary materials

## Data Availability

All relevant data that support the findings of this study are available from the corresponding author upon reasonable request. Full and uncropped western blots are in the supplemental materials.
