# Supplementary material for: The E3 ubiquitin ligase TRIM56 promotes aggregation and activation of Src protein through Lys63-linked polyubiquitination in hepatocellular carcinoma
Source: Cell Death Dis. 2025 Oct 16;16(1):731. doi: 10.1038/s41419-025-08074-1 (PMC12533068; doi:10.1038/s41419-025-08074-1)
Supplement: Supplementary file 1 — Supplementary materials [file 41419_2025_8074_MOESM1_ESM.docx]

**Title page**

**The E3 ubiquitin ligase TRIM56 promotes aggregation and activation of Src protein through Lys63-linked polyubiquitination in hepatocellular carcinoma**

Lihui Zhu^1,2,3^, Xiuling Cui^1,3,^ Hongwei Xu^2^, Min Yang^1^, Lihui Han^1^*

1. Department of Immunology, Shandong Provincial Key Laboratory of Infection & Immunology, Shandong University School of Basic Medical Sciences, Jinan, Shandong, 250012, China.
2. Department of Gastroenterology, Shandong Provincial Hospital Affiliated to Shandong First Medical University, Jinan, Shandong, 250021, China.

3 Lihui Zhu and Xiuling Cui contributed equally to this work.

* Corresponding Author: Lihui Han, M.D., Ph.D., [hanlihui@sdu.edu.cn,](mailto:hanlihui@sdu.edu.cn,) Department of Immunology, Shandong University School of Basic Medical Sciences, 44 Wenhua Xi Road, Jinan, Shandong, 250012, China.

**Running title：TRIM56 promotes aggregation and activation of Src in HCC**

**Supplementary Figures**

**Supplementary Figure 1**


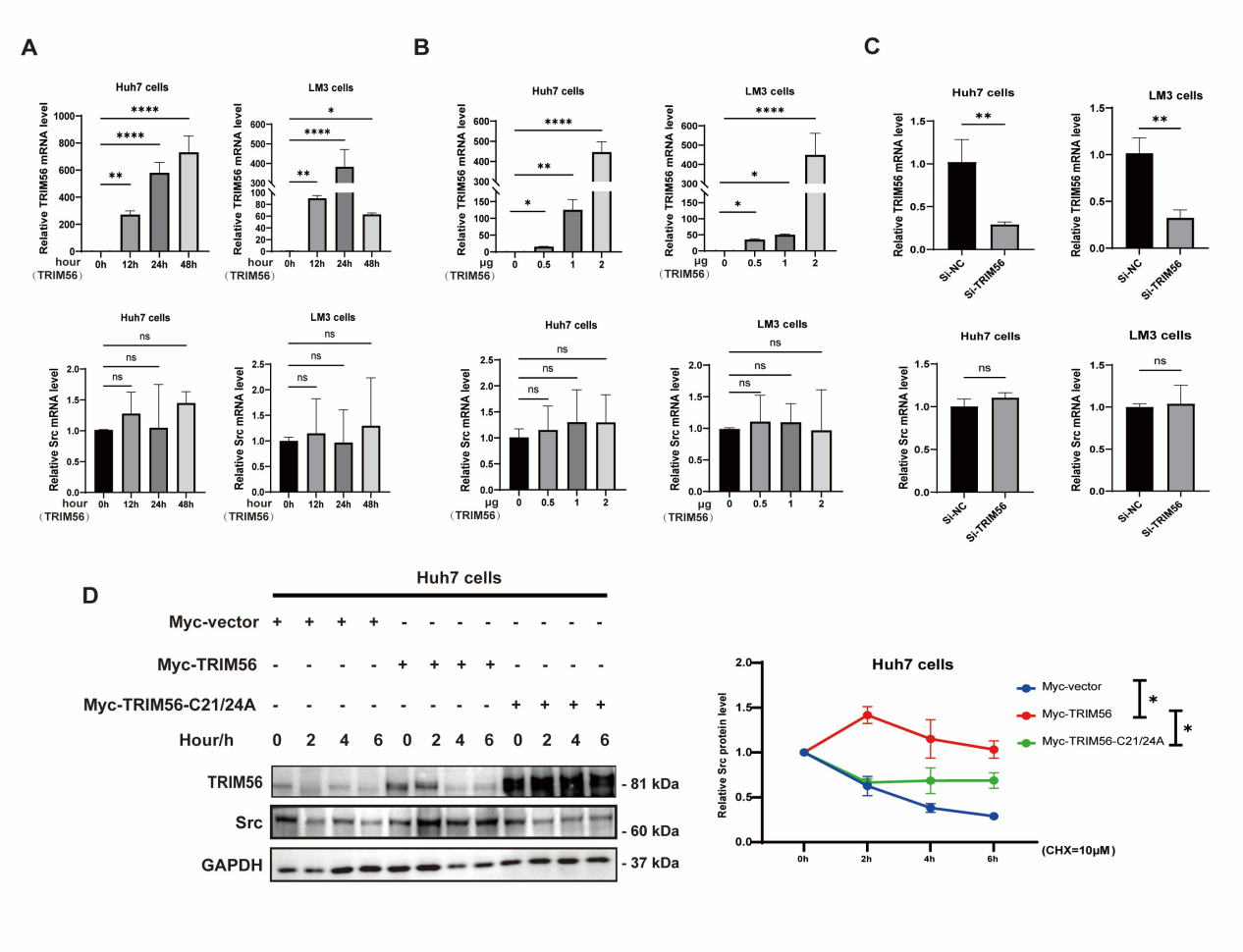


**Supplementary Figure 1. TRIM56 promoted the activation of Src at the protein level via maintaining the protein stability of Src.**

**(A)** HCC cells were transfected with the Myc-TRIM56, the mRNA levels of TRIM56 and Src were detected by real-time PCR assay at the indicated time points. **(B)** Real-time PCR analysis of the mRNA levels of TRIM56 and Src in HCC cells transfected with different dosages of Myc-TRIM56 plasmids. **(C)** Real-time PCR analysis of the mRNA levels of TRIM56 and Src in HCC cells transfected with SiRNA against TRIM56(Si-TRIM56), or its nonsense control(Si-NC). **(D)** Huh7 cells were transfected with Myc-TRIM56 plasmid or the enzymatic activity-dead (C21/24A) TRIM56 mutant plasmids, and the cells were treated with cyclohexamide (CHX) for 0h, 2h, 4h and 6h before western blot assay of the Src protein(left panel). Band intensities of the key proteins were further quantitatively analyzed in the indicated groups(right panel). ns > 0.05, *P < 0.05, **P < 0.01, and ****P< 0.0001 (One-way ANOVA) for the statistical analysis of the indicated groups,error bar are mean±SD. The presented figures are representative data from at least three independent experiments.

**Supplementary Figure 2**


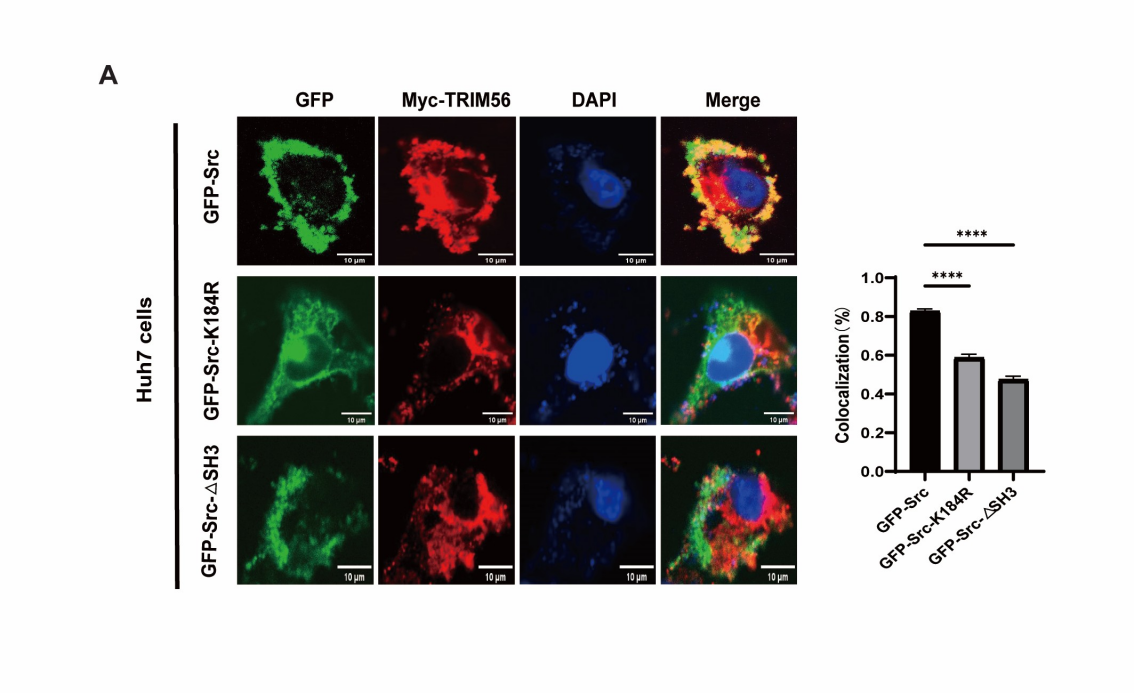


**Supplementary Figure 2. Mutations in Src resulted in a diminished interaction between Src and TRIM56.**

**(A)**Huh7 cells were transfected with Myc-TRIM56 plasmid together with GFP-Src, or GFP-Src-K184R mutant or GFP-Src-ΔSH3 mutant.Then the cells were fixed and stained with primary antibodies against GFP or Myc followed by staining with the fluorescence-conjugated secondary antibodies. DAPI was used to stain the nuclei, and the colocalization of TRIM56 and Src was visualized as yellow fluorescence in the merged panel, scale bar, 10 μm, *****P* < 0.0001 (Student’s *t* test) for statistical analysis of the indicated groups , error bar are mean±SD.
